# 

**Published:** 1879-03

**Authors:** 


PAMPHLETS RECEIVED. — The Brazillian Tea (Cha Mate), erroneously called Paraguay Tea. By Chas. Wm. Zaremba, M.D., member of the Academy of Natural Sciences, Philadelphia, etc., etc.

Report of investigations into the Pathology of Diphtheria, conducted by Edward Curtis, M.D, and Thos. E. Satterthwaite, M.D., N. Y. 1877.

Circular of information relating to the Pennsylvania Training School for Feeble-Minded Children. Westchester, Pa.

Proceedings of the Medical Society of the county of Kings. Monthly report. Brooklyn, N. Y.

The first annual report of the Presbyterian Eye and Ear Charity Hospital, No. 77 E. Baltimore street, Baltimore, Md. 1878.

Twenty-sixth annual announcement Medical Department University of Vermont. 1879.

Memorandum of the American Public Health Association on legislation affecting the public health.

Report of the Special Relief Committee, I. O. O. F., of Memphis, Tenn. 1878.

Transactions of the Detroit Medical and Library Association. January, 1879.

Bulletin of the Public Health, issued by the Surgeon General U. S. Marine Hospital Service, under the National Quarantine Act of 1878.

### INDEX TO VOLUME XVI.

PAGE.

Absence of the Radius........ 703
An Anomaly in Obstitrics..... 584
Anomalous Twin Pregnancy.. 763
Action of the Tinctnre of Iodine upon the Uterine Neck. 512
Atlanta Medical College Clinic. 466
American Pharmaceutical Association.................. 506
Acute Bright's Disease cured by Jaborandi................. 508
American Pnarmaceutical Association. Address of President Saunders............. 513
A Large Heart................ 445
Action of Purgatives.......... 447
Advantages of Legal Control over Prostitution.......... 426
American Medical Association 169, 235
Amputation of the Cervix Uteri. 441
Accidental Hemorrhage, treatment of.................. 355
An Essay..................... 397
Anatomy..................... 436
Abcess at the Verge of the Anus...................... 440
Antagonism of Therapeutic Agents...................... 436
Atlanta Academy of Medicine, 79, 130, 407, 454, 534, 593, 647, 731
Atlanta Medical College, 429, 694, 754
A Man who Burst............ 382
Action of Remedies.......... 8
Atlanta Medico-Chirurgical Society...................... 23
Ague, Pathology of, and the Agent that Produces it..... 32
Abortion, case of............. 48
Albuminuria During Pregnancy...................... 62
Acne, Local Treatment of.... 192
Anal Fissure, Carron Oil in... 187
A Good Cleansing Fluid...... 192
A Peculiar Deformity........ 196
A Remarkable Case.......... 640
A Few Days' Obstetrical Experience..................... 645
Alimentation in Health and Disease..................... 655

PAGE.

Anæsthesia Cured by the Application of Metal............ 702
Brain, Duality of............. 110
Blennorrhagic Affections, treatment of................... 335
Bromide of Potassium Externally as a Caustic and Hæmostatic..................... 439
Botany in its Relations with Medicine, No. 5............ 1
Boils, Arnica as a Remedy for. 125
Baltimore Academy of Medicine....................... 197
Baltimore Medical Association 83
Baltimore Clinical Society.... 85
Clinical History of the Medical and Surgical Diseases of Women....................... 506
Cardiac Embolism Following Delivery................... 499
Chronic Chloral Poisoning.... 495
Cerebro-Spinal Meningetis.... 451
Cysticerci in the Brain Diagnosticated During Life...... 704
Congenital Deformity of the Superior Extremity......... 701
Croup and Diphtheria, German Treatment of.............. 700
Correspondence.............. 641
Chloral as a Counter-Irritant.. 639
Criminal Abortion............ 165
Coming Duties of the Accoucher..................... 190
Conspectus of Organic Materia Medica and Pharmacal Botany........................ 628
Consolidation of Medical Colleges...................... 443
Carbolic Acid in Piles, Objections to Use of............ 185
Case of Extra-Uterine Pregnancy – Death................ 611
Constipation................. 128
Chloral Hydrate as an Antiseptic...................... 128
Cancer, Treatment of by Bromine...................... 439
Catalepsy Produced by a Hair. 439
Consultation with Homœopaths and the use of their Medicines.................. 423

PAGE.

Can Syphilis be Transmitted by Means of the Spermatic Fluid? .......... 255
Congress on Legal Medicine .. 446
Clinical Lecture .......... 17
Causes of Suicide .......... 443
Conservative Surgery ....... 67
Coughs, Carbolic Acid Spray in 254
Cleanly Liniment .......... 191
Case of Intestinal Obstruction by a Deverticulum Verum of Meckel .......... 215
Damiana .......... 761
Detroit Academy of Medicine. 748
Difficult Labor in a case of Twins .......... 730
Dysentery, Chloral in .......... 5[illegible]7
Diphtheria .......... 532
Diphtheria .......... 64
Diphtheria .......... 760
Diarrhœa, Treatment of by Oxide of Zinc .......... 512
Diphtheria, Analagous to Erysipulatous Inflammation ... 530
Does Quinine Affect the Hearing? .......... 638
Dr. Walker's Modification of Sayre's Apparatus .......... 632
Differential Diagnosis .......... 628
Diphtheria, Treatment of ..... 189
Distinctive Lists of an Insane and Sane Delusion .......... 223
Diaphragmatic Hernia .......... 145
Delirium Tremens, Treatment of .......... 127
Diphtheria—its Nature and Treatment .......... 695
Double Fetation .......... 116
Diphtheria, Observations on .. 96
Diphtheria, Treatment of by the French School .......... 383
Diabetes, Treatment of by Salacine .......... 440
Damiana as a Nerve Tonic .... 696
Dysmenorrhœa, its Treatment. 346
Daltonism — Treatment of by Fuchsine .......... 373
Erectile Tumors, Treatment of 61
Epilepsy, Electricity in Treatment of .......... 103
Erysipelas, Silicate of Soda in. 125
Epilepsy, Treatment of ....... 186
Epilepsy, Atropia in .......... 316
Enlarged Prostate, Treatment of .......... 317
Essex North District Medical Society .......... 605
Eff cts of Rhubarb and Santonine on the Urine .......... 635

PAGE.

Essay on Hydrophobia ....... 631
Effect of Bicarbonate of Potash on the Acidity of the Urine .. 636
Elementary Lessons in Electricity .......... 688
Essentials of Chemistry, Organic and Inorganic .......... 571
Epistaxis Arrested by Subcutaneous Injections of Ergotine 576
Eczema, Local Treatment of .. 471
Editorial Correspondence . . 181
183, 250, 758
Eye Disease, Eserine and Pilocarpine in Treatment of ..... 42
Elephantiasis—A New Tinea ... 418
Eye-sight, Seven Good Rules for Preserving .......... 377
Elastic Adhesive Plaster ....... 446
European Correspondence .... 169
245, 429, 565
Foreign Bodies in Ear and Nose 714
For Flutulent Dyspepsia .. .. 191
Fown's Manual of Chemistry .. 311
Gynæcological Society of Boston .......... 212
Gastric Ulcer .......... 225, 548
How to Kill a Tape-worm in an Hour .......... 576
How to Make a Poultice ...... 640
How to Cough .......... 764
Hydrophobia Cured by Oxygen 639
Hydrophobia, Jaborandi as a Remedy in .......... 127
Heart, Neurosal Affections of .. 50
Hay Fever .......... 440
Hints on the Hypodermic Use of Morphia .......... 228
Hydrocele, Treatment of ...... 318
Ipecac as a Hæmostatic ....... 442
Irritable Testis .......... 420
Introductory Lecture .......... 385
In Memoriam — Dr. Charles Rauschenberg .......... 757
Index to Original Communications, etc .......... 759
Insanity, Causes of in the United States .......... 98
Intra-Uterine Pregnancy, etc .. 113
Invisible Ink for Postals ...... 190
Indian Obstetrics .......... 313
Is the Hypodermic Injection of Piles Dangerous? .......... 607
Innervation of the Uterus and of its Vessels .......... 634
Inversion of the Uterus ....... 680
Icteric Urine .......... 511
Intra-Ocular Circulation: Rhythmical Changes in the Venous Pulse of the Optic Disk 483

PAGE.

Illustrirte Vierteljahrsschrift der Ærztlichen Polytechnick 507
Intra-Uterine Medication in Sterility 751
Jaborandi and its Active Principle Pilocarpine 231
Justice Rendered 253
Leprosy, Notes on Cases 674
Laparo-Elytriotomy 669
Lactopeptine 318, 426
Laceration of the Cervix a Cause of Steriliay, Miscarriage, etc 762
Lumbago, Salicylic Acid in 101
Lead Poisoning 381
Meteorological Report for November 566
Medical Societies in the Country 577
Mistletoe as an Oxytoxic 150
Meningitis 427
Milk as a Vehicle for Quinine 447
Membranous Dysmenorrhœa 256
Manual of Operative Surgery 311
Medical Clinic 415
Medical Use of the Telephone 255
Medical Associations 693
McDowell Monument 381
Medical College Association 241
Medical Association of Georgia 120, 253
Metric System 373
Metric System in the United States Army 188
Miner's Wrist 213
Medical and Surgical Society of Baltimore 206
Metric System in Medicine 231
New Method of Covering the Taste of Cod-Liver Oil 638
Nervous Diseases 311
Night-Sweating, External Use of Tincture of Belladonna in 187
New York Academy of Medicine 28
Operation for Stricture followed by Uræmic Poisoning 449
On the Treatment of the Various Forms of Acne and of Rosacea 571
On Rest and Pain 571
Obstinate Vomiting Cured by Meat-Pancreas Injection 701
Official or Officinal? 378
Ovariotomy, Case of 348
Opium Habit 626
Opium Habit, Cure of 643
Opium Habit and Amyl Nitrite 761
Pamphlets Received 760

PAGE.

Proceedings of Boston Society for Medical Observation 743
Proceedings New Orleans Medical Society 746
Poison in the Nursery 188
Professional Secrets 189
Public Analysts 190
Pneumatic Forceps 316
Principles of Medical Practice, Formerly and Now 329
Progress of Gynæcology 302
Pathological Society of Philadelphia 594
Philadelphia County Medical Society 597
Placenta Prævia 620
Perfumed Solution of Iodoform 640
Pressure in Treatment of Uterine Diseases 697
Pessaries 511
Procidentia Uteri and its Treatment 537
Proceedings Suffolk Medical Society 456
Philadelphia Pathological Society 461
Piles, Carbolic Acid in 101
Physiology, Recent Progress in 139
Puerperal Eclampsia 65, 129
Prescription Writing 185
Prostatic Derangement 6
Premature Baldness 391
Poisoning by Oil of Bitter Almonds 444
Phimosis as a Cause of Rupture in Children 381
Penneorraphy 349
Quarantine 425
Quinine Exanthem 63
Quinia Pills 126
Questions of Maternal Influence 509
Recent Progress in Pathology and Pathological Anatomy 543
Rhode Island Medical Society 604
Rheumatoid Inflammation of the Joints of Women 217
Rapid Dilatation of the Female Urethra for the Relief of Vesical Irritability 153
Rotheln, Rubeola — German Measles 135
Salicylic Acid as an Anaphrodisiac 441
Santonine Poisoning 127
Substitute for the Mother's Milk During Infancy 146
Smith, Prof. Francis G., Death of 190

PAGE.

Strictures of the Cervical Canal and of the Internal and External Os.................... 257
Substitute for Calomel........ 315
Sunstroke.......................... 316
Some Points in Regard to the Actions and Uses of Pilocarpin and its Salts............ 609
Skin Diseases—Notes on the Treatment of............... 629
Suggestions for Sleeplessness. 699
Syphilitic Milkmen........... 699
Some Thoughts on the Therapeutics of Parturition....... 487
Skin Grafting, use of Common Warts of the hand in......... 354
Skin Grafting in the Colored Races....................... 438
Skin Grafting en Masse........ 762
Seborrhœa, Pathology of..... 89
Spencer Wells' Ovariotomies.. 443
Specialists, French Criticism of 62
Skin Diseases, Atlas of....... 123
Strychnine in Cough Mixtures. 124
Skin Diseases, Substitute for Cod Liver Oil in............ 118
Transfusion..................436, 574
Tapeworm, New Treatment of. 437
Therapeutic Value of Nitrate of Lead.................... 437
Threads of Whale Tendon as a Substitute for Cat-gut...... 441
The Usefulness of Cremation.. 442
The Dietetic Value of Light Wines......................... 444
The Great Physical Forces, New and Original Theories of 760
Treatment of Bromide Eruption........................ 426
To Keep the Cord from Slipping in Extracting the Placenta......................... 191
Typhoid Fever................ 197
The Science and Art of Surgery 310
Thymal, the New Antiseptic.. 319
The Physiology of Sleep...... 618
Turpentine Vapor in Accidents from Chloroform Vapor .... 639
The Thermal Death Point of Septic Organisms.......... 510

PAGE.

Tabes Mesenterica............ 589
The Man-Fish of Tennessee... 572
The Eucalyptus in Malarial Disorders................... 575
Tansy in Pruritis Vulvæ...... 576
The Theory of Reproduction.. 490
The Yellow Fever Commission. 505
The International Decimal System of Weights and Measures.......................... 504
The Human Eye—An Essay.. 705
The Periodicity of Epidemics.. 384
Treatise on Surgical Diseases and Injuries ............... 435
The Chinese Question in its Medical Bearings .......... 375
Traumatic Tetanus Treated by Physastigma ............. 344
Travel in Phthisis............. 380
Unusual Effects of Quinine.... 554
Uterine Trouble and its Treatment.................... 193
Ulceration of Frænum Linguæ in Pertussis................ 638
Venesection as an Anti-Hemorrhagic...................... 508
Vomiting of Pregnancy and its Treatment................. 156
Vicarious Menstruation, Case of.......................... 152
Valedictory Addresses..69, 73, 716, 724
Venereal Sores—Iodoform.... 382
Volume XVI................. 60
Why the Philadelphia County Medical Society is a Secret Society.................... 634
Whooping-Cough, Nitric Acid for........................ 188
Wounds.................... 383
Yellow Fever, Microscopical Observations in ... .... 480
Yellow Fever Commission.... 558
Yellow Fever, Form and Contagiousness of.............. 637
Yellow Fever Board of Experts....................... 624
Yellow Fever................ 308
Yellow Fever, Preventive Treatment of in Havana.... 341

